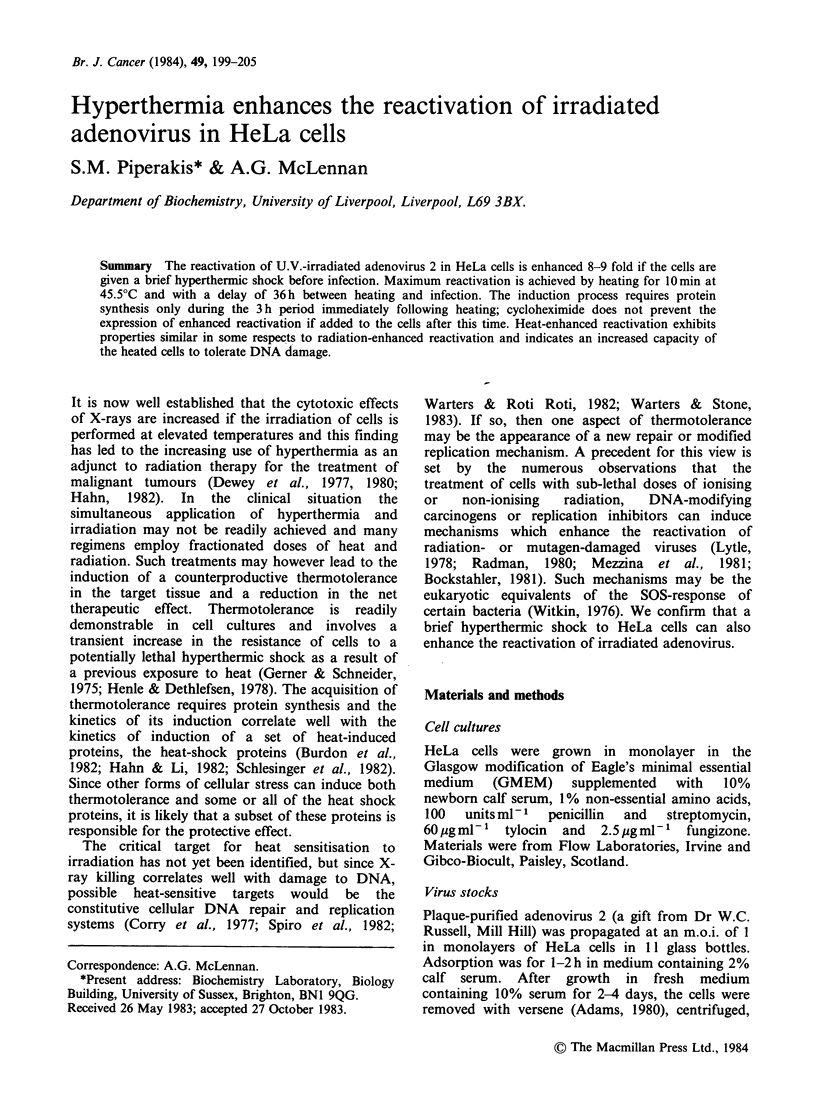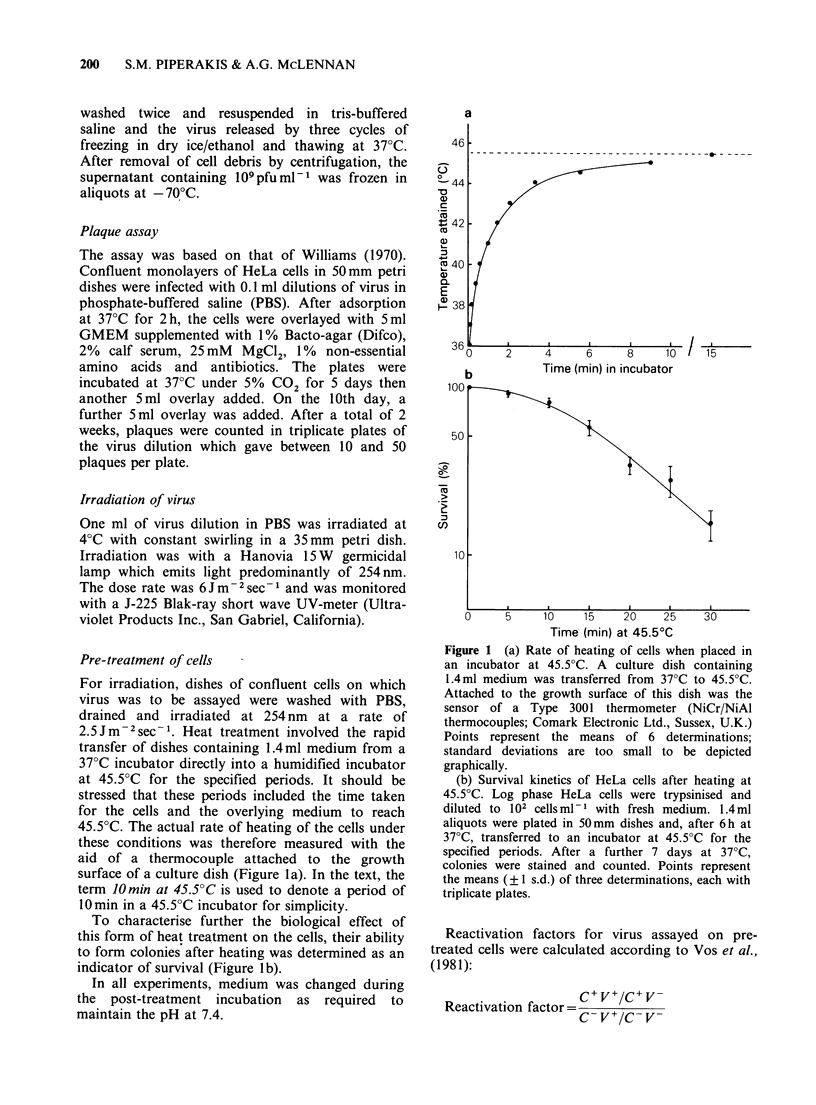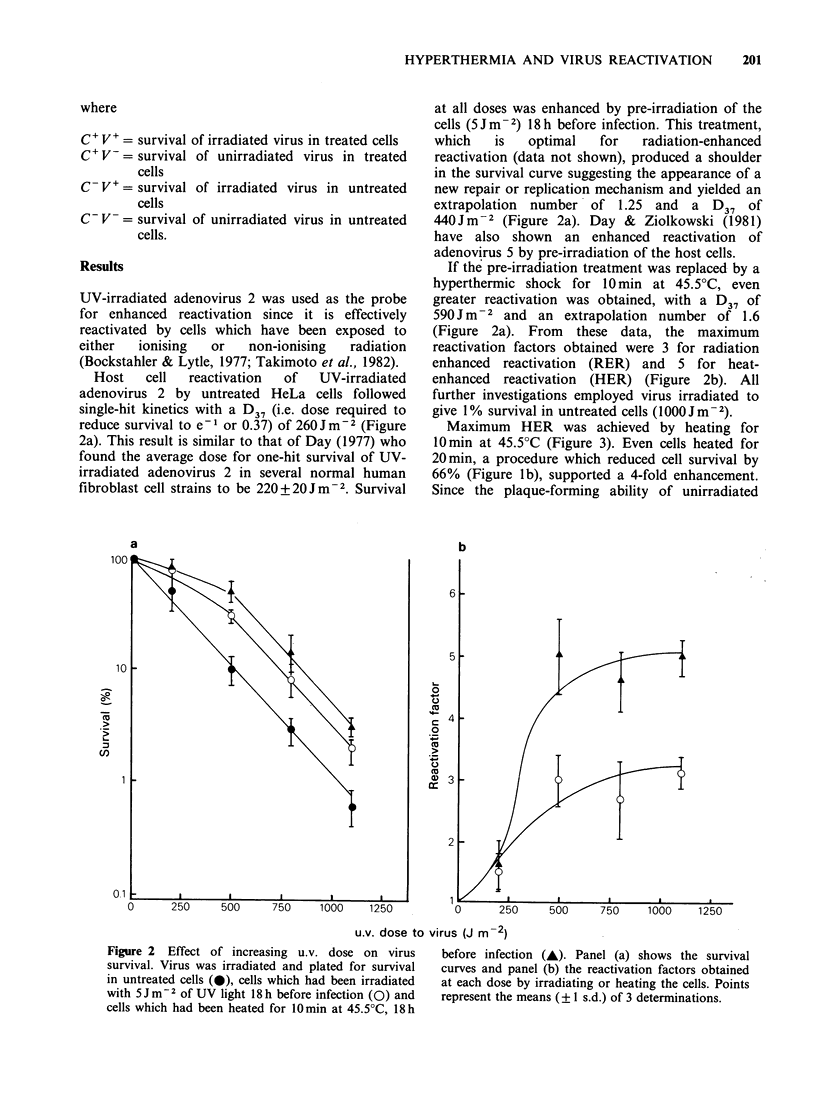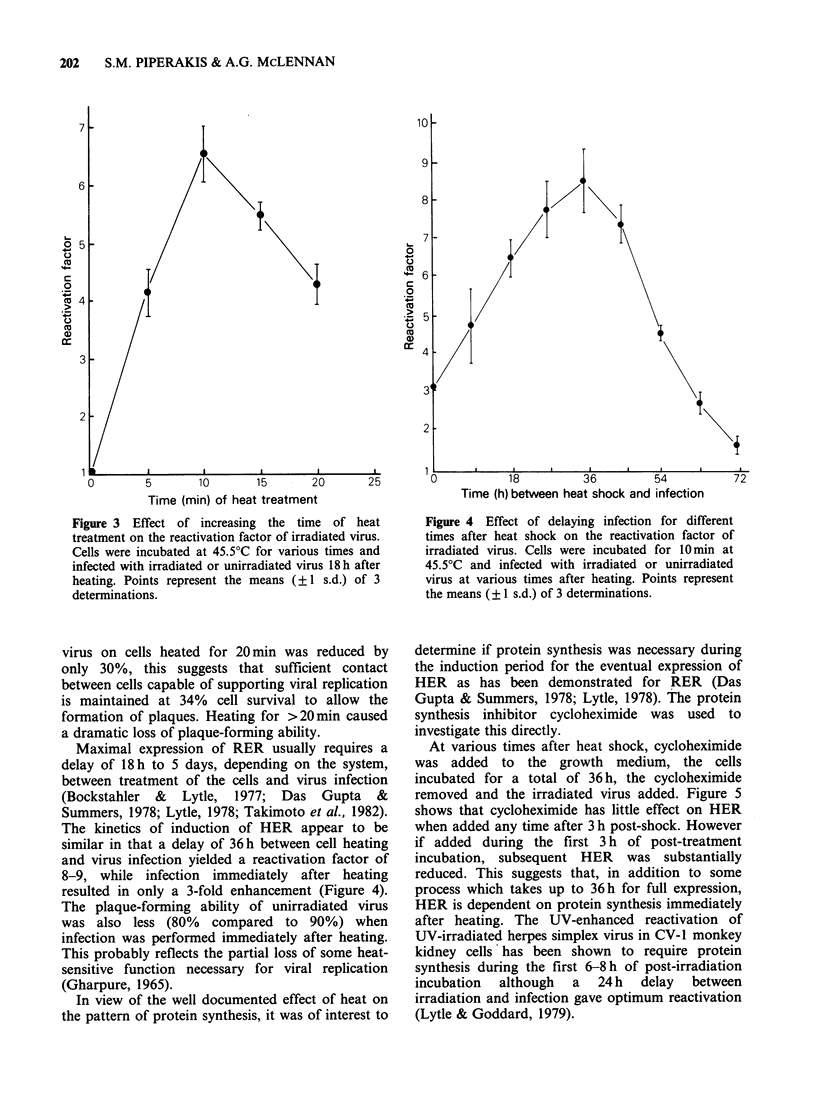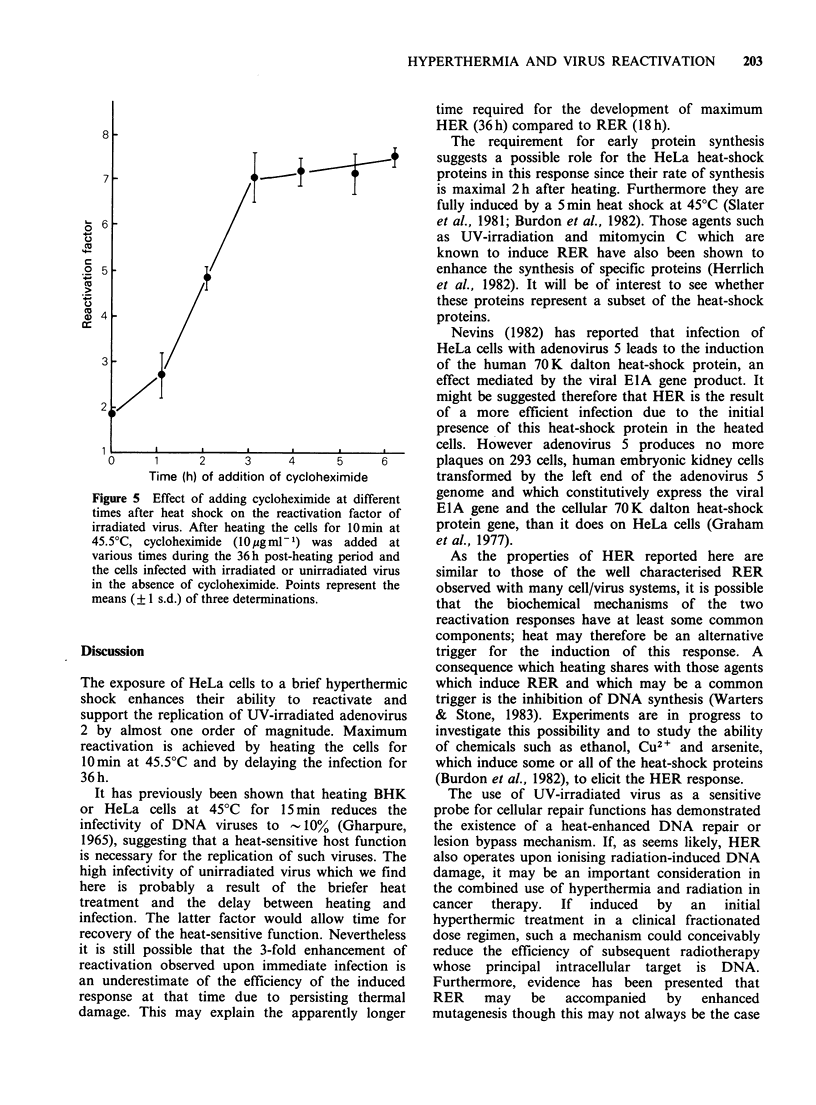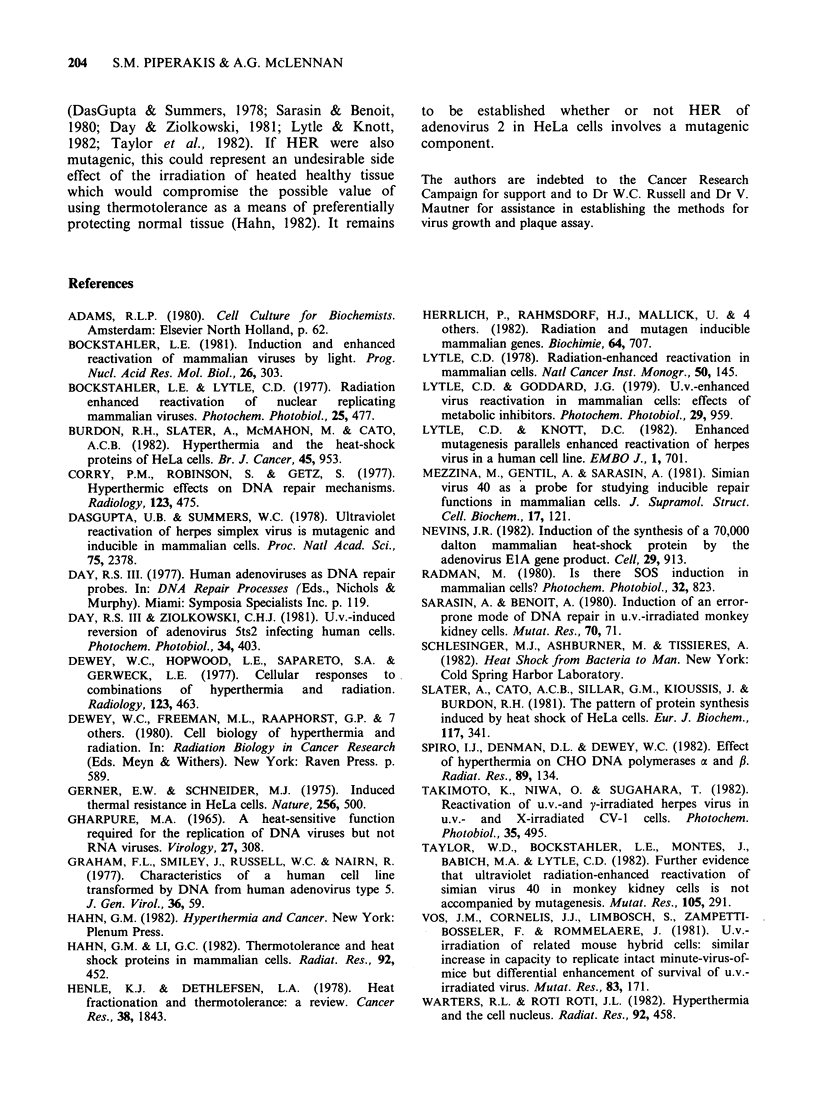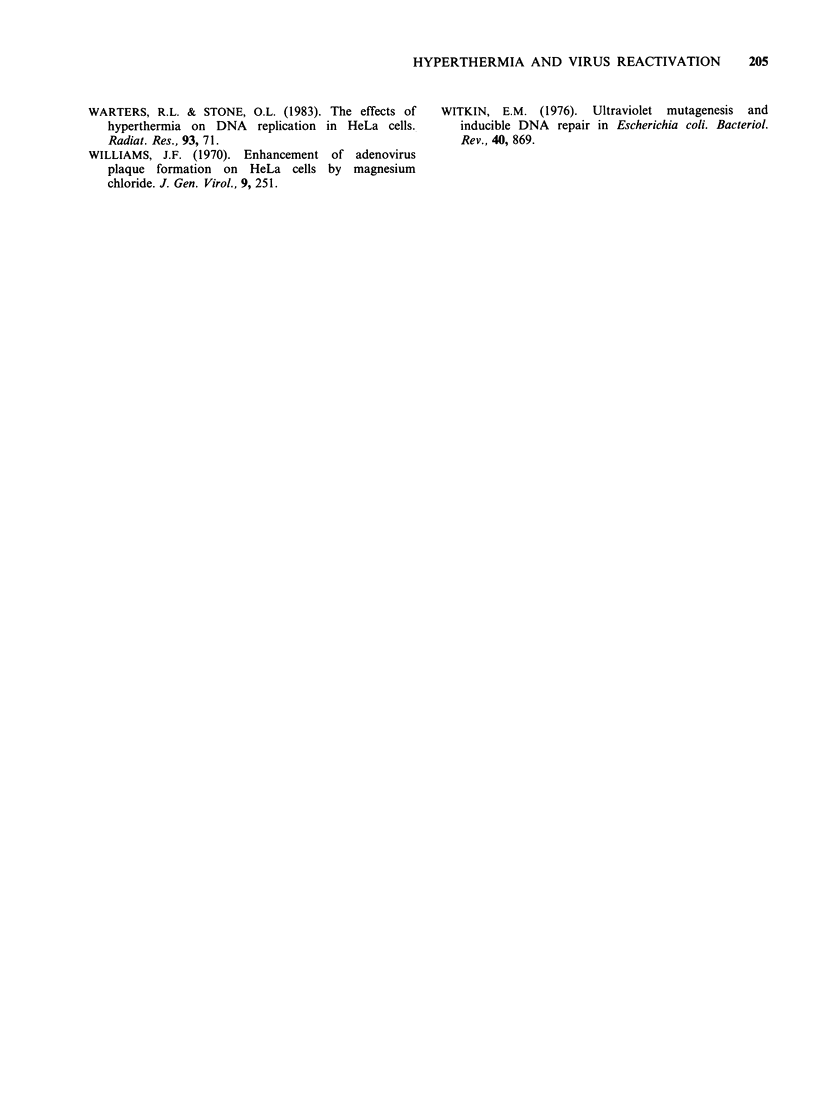# Hyperthermia enhances the reactivation of irradiated adenovirus in HeLa cells.

**DOI:** 10.1038/bjc.1984.32

**Published:** 1984-02

**Authors:** S. M. Piperakis, A. G. McLennan

## Abstract

The reactivation of U.V.-irradiated adenovirus 2 in HeLa cells is enhanced 8-9 fold if the cells are given a brief hyperthermic shock before infection. Maximum reactivation is achieved by heating for 10 min at 45.5 degrees C and with a delay of 36 h between heating and infection. The induction process requires protein synthesis only during the 3 h period immediately following heating; cycloheximide does not prevent the expression of enhanced reactivation if added to the cells after this time. Heat-enhanced reactivation exhibits properties similar in some respects to radiation-enhanced reactivation and indicates an increased capacity of the heated cells to tolerate DNA damage.


*Br. J. Cancer* (1984), **49**, 199–205

### Hyperthermia enhances the reactivation of irradiated adenovirus in HeLa cells

S.M. Piperakis* & A.G. McLennan

*Department of Biochemistry, University of Liverpool, Liverpool, L69 3BX.*

**Summary** The reactivation of U.V.-irradiated adenovirus 2 in HeLa cells is enhanced 8–9 fold if the cells are given a brief hyperthermic shock before infection. Maximum reactivation is achieved by heating for 10 min at 45.5°C and with a delay of 36 h between heating and infection. The induction process requires protein synthesis only during the 3 h period immediately following heating; cycloheximide does not prevent the expression of enhanced reactivation if added to the cells after this time. Heat-enhanced reactivation exhibits properties similar in some respects to radiation-enhanced reactivation and indicates an increased capacity of the heated cells to tolerate DNA damage.

It is now well established that the cytotoxic effects of X-rays are increased if the irradiation of cells is performed at elevated temperatures and this finding has led to the increasing use of hyperthermia as an adjunct to radiation therapy for the treatment of malignant tumours (Dewey *et al.*, 1977, 1980; Hahn, 1982). In the clinical situation the simultaneous application of hyperthermia and irradiation may not be readily achieved and many regimens employ fractionated doses of heat and radiation. Such treatments may however lead to the induction of a counterproductive thermotolerance in the target tissue and a reduction in the net therapeutic effect. Thermotolerance is readily demonstrable in cell cultures and involves a transient increase in the resistance of cells to a potentially lethal hyperthermic shock as a result of a previous exposure to heat (Gerner & Schneider, 1975; Henle & Dethlefsen, 1978). The acquisition of thermotolerance requires protein synthesis and the kinetics of its induction correlate well with the kinetics of induction of a set of heat-induced proteins, the heat-shock proteins (Burdon *et al.*, 1982; Hahn & Li, 1982; Schlesinger *et al.*, 1982). Since other forms of cellular stress can induce both thermotolerance and some or all of the heat shock proteins, it is likely that a subset of these proteins is responsible for the protective effect.

The critical target for heat sensitisation to irradiation has not yet been identified, but since X-ray killing correlates well with damage to DNA, possible heat-sensitive targets would be the constitutive cellular DNA repair and replication systems (Corry *et al.*, 1977; Spiro *et al.*, 1982; Warters & Roti Roti, 1982; Warters & Stone, 1983). If so, then one aspect of thermotolerance may be the appearance of a new repair or modified replication mechanism. A precedent for this view is set by the numerous observations that the treatment of cells with sub-lethal doses of ionising or non-ionising radiation, DNA-modifying carcinogens or replication inhibitors can induce mechanisms which enhance the reactivation of radiation- or mutagen-damaged viruses (Lytle, 1978; Radman, 1980; Mezzina *et al.*, 1981; Bockstahler, 1981). Such mechanisms may be the eukaryotic equivalents of the SOS-response of certain bacteria (Witkin, 1976). We confirm that a brief hyperthermic shock to HeLa cells can also enhance the reactivation of irradiated adenovirus.

Correspondence: A.G. McLennan.
*Present address: Biochemistry Laboratory, Biology Building, University of Sussex, Brighton, BN1 9QG.
Received 26 May 1983; accepted 27 October 1983.

### Materials and methods

### *Cell cultures*

HeLa cells were grown in monolayer in the Glasgow modification of Eagle's minimal essential medium (GMEM) supplemented with 10% newborn calf serum, 1% non-essential amino acids, 100 units $ml^{-1}$ penicillin and streptomycin, 60 $\mu g\, ml^{-1}$ tylocin and 2.5 $\mu g\, ml^{-1}$ fungizone. Materials were from Flow Laboratories, Irvine and Gibco-Biocult, Paisley, Scotland.

### *Virus stocks*

Plaque-purified adenovirus 2 (a gift from Dr W.C. Russell, Mill Hill) was propagated at an m.o.i. of 1 in monolayers of HeLa cells in 1 l glass bottles. Adsorption was for 1–2 h in medium containing 2% calf serum. After growth in fresh medium containing 10% serum for 2–4 days, the cells were removed with versene (Adams, 1980), centrifuged,

© The Macmillan Press Ltd., 1984

washed twice and resuspended in tris-buffered saline and the virus released by three cycles of freezing in dry ice/ethanol and thawing at 37°C. After removal of cell debris by centrifugation, the supernatant containing $10^9$ pfu ml$^{-1}$ was frozen in aliquots at −70°C.

*Plaque assay*

The assay was based on that of Williams (1970). Confluent monolayers of HeLa cells in 50 mm petri dishes were infected with 0.1 ml dilutions of virus in phosphate-buffered saline (PBS). After adsorption at 37°C for 2 h, the cells were overlayed with 5 ml GMEM supplemented with 1% Bacto-agar (Difco), 2% calf serum, 25 mM $MgCl_2$, 1% non-essential amino acids and antibiotics. The plates were incubated at 37°C under 5% $CO_2$ for 5 days then another 5 ml overlay added. On the 10th day, a further 5 ml overlay was added. After a total of 2 weeks, plaques were counted in triplicate plates of the virus dilution which gave between 10 and 50 plaques per plate.

*Irradiation of virus*

One ml of virus dilution in PBS was irradiated at 4°C with constant swirling in a 35 mm petri dish. Irradiation was with a Hanovia 15 W germicidal lamp which emits light predominantly of 254 nm. The dose rate was 6 J m$^{-2}$ sec$^{-1}$ and was monitored with a J-225 Blak-ray short wave UV-meter (Ultraviolet Products Inc., San Gabriel, California).

*Pre-treatment of cells*

For irradiation, dishes of confluent cells on which virus was to be assayed were washed with PBS, drained and irradiated at 254 nm at a rate of 2.5 J m$^{-2}$ sec$^{-1}$. Heat treatment involved the rapid transfer of dishes containing 1.4 ml medium from a 37°C incubator directly into a humidified incubator at 45.5°C for the specified periods. It should be stressed that these periods included the time taken for the cells and the overlying medium to reach 45.5°C. The actual rate of heating of the cells under these conditions was therefore measured with the aid of a thermocouple attached to the growth surface of a culture dish (Figure 1a). In the text, the term *10 min at 45.5°C* is used to denote a period of 10 min in a 45.5°C incubator for simplicity.

To characterise further the biological effect of this form of heat treatment on the cells, their ability to form colonies after heating was determined as an indicator of survival (Figure 1b).

In all experiments, medium was changed during the post-treatment incubation as required to maintain the pH at 7.4.

**Figure 1** (a) Rate of heating of cells when placed in an incubator at 45.5°C. A culture dish containing 1.4 ml medium was transferred from 37°C to 45.5°C. Attached to the growth surface of this dish was the sensor of a Type 3001 thermometer (NiCr/NiAl thermocouples; Comark Electronic Ltd., Sussex, U.K.) Points represent the means of 6 determinations; standard deviations are too small to be depicted graphically.

(b) Survival kinetics of HeLa cells after heating at 45.5°C. Log phase HeLa cells were trypsinised and diluted to $10^2$ cells ml$^{-1}$ with fresh medium. 1.4 ml aliquots were plated in 50 mm dishes and, after 6 h at 37°C, transferred to an incubator at 45.5°C for the specified periods. After a further 7 days at 37°C, colonies were stained and counted. Points represent the means ($\pm 1$ s.d.) of three determinations, each with triplicate plates.

Reactivation factors for virus assayed on pre-treated cells were calculated according to Vos *et al.*, (1981):

$$\text{Reactivation factor} = \frac{C^+V^+/C^+V^-}{C^-V^+/C^-V^-}$$

where

$C^+V^+$ = survival of irradiated virus in treated cells
$C^+V^-$ = survival of unirradiated virus in treated cells
$C^-V^+$ = survival of irradiated virus in untreated cells
$C^-V^-$ = survival of unirradiated virus in untreated cells.

### Results

UV-irradiated adenovirus 2 was used as the probe for enhanced reactivation since it is effectively reactivated by cells which have been exposed to either ionising or non-ionising radiation (Bockstahler & Lytle, 1977; Takimoto *et al.*, 1982).

Host cell reactivation of UV-irradiated adenovirus 2 by untreated HeLa cells followed single-hit kinetics with a $D_{37}$ (i.e. dose required to reduce survival to $e^{-1}$ or 0.37) of 260 J m$^{-2}$ (Figure 2a). This result is similar to that of Day (1977) who found the average dose for one-hit survival of UV-irradiated adenovirus 2 in several normal human fibroblast cell strains to be 220±20 J m$^{-2}$. Survival at all doses was enhanced by pre-irradiation of the cells (5 J m$^{-2}$) 18 h before infection. This treatment, which is optimal for radiation-enhanced reactivation (data not shown), produced a shoulder in the survival curve suggesting the appearance of a new repair or replication mechanism and yielded an extrapolation number of 1.25 and a $D_{37}$ of 440 J m$^{-2}$ (Figure 2a). Day & Ziolkowski (1981) have also shown an enhanced reactivation of adenovirus 5 by pre-irradiation of the host cells.

If the pre-irradiation treatment was replaced by a hyperthermic shock for 10 min at 45.5°C, even greater reactivation was obtained, with a $D_{37}$ of 590 J m$^{-2}$ and an extrapolation number of 1.6 (Figure 2a). From these data, the maximum reactivation factors obtained were 3 for radiation enhanced reactivation (RER) and 5 for heat-enhanced reactivation (HER) (Figure 2b). All further investigations employed virus irradiated to give 1% survival in untreated cells (1000 J m$^{-2}$).

Maximum HER was achieved by heating for 10 min at 45.5°C (Figure 3). Even cells heated for 20 min, a procedure which reduced cell survival by 66% (Figure 1b), supported a 4-fold enhancement. Since the plaque-forming ability of unirradiated

**Figure 2** Effect of increasing u.v. dose on virus survival. Virus was irradiated and plated for survival in untreated cells (●), cells which had been irradiated with 5 J m$^{-2}$ of UV light 18 h before infection (○) and cells which had been heated for 10 min at 45.5°C, 18 h before infection (▲). Panel (a) shows the survival curves and panel (b) the reactivation factors obtained at each dose by irradiating or heating the cells. Points represent the means (±1 s.d.) of 3 determinations.

**Figure 3** Effect of increasing the time of heat treatment on the reactivation factor of irradiated virus. Cells were incubated at 45.5°C for various times and infected with irradiated or unirradiated virus 18 h after heating. Points represent the means (±1 s.d.) of 3 determinations.

**Figure 4** Effect of delaying infection for different times after heat shock on the reactivation factor of irradiated virus. Cells were incubated for 10 min at 45.5°C and infected with irradiated or unirradiated virus at various times after heating. Points represent the means (±1 s.d.) of 3 determinations.

virus on cells heated for 20 min was reduced by only 30%, this suggests that sufficient contact between cells capable of supporting viral replication is maintained at 34% cell survival to allow the formation of plaques. Heating for >20 min caused a dramatic loss of plaque-forming ability.

Maximal expression of RER usually requires a delay of 18 h to 5 days, depending on the system, between treatment of the cells and virus infection (Bockstahler & Lytle, 1977; Das Gupta & Summers, 1978; Lytle, 1978; Takimoto *et al.*, 1982). The kinetics of induction of HER appear to be similar in that a delay of 36 h between cell heating and virus infection yielded a reactivation factor of 8–9, while infection immediately after heating resulted in only a 3-fold enhancement (Figure 4). The plaque-forming ability of unirradiated virus was also less (80% compared to 90%) when infection was performed immediately after heating. This probably reflects the partial loss of some heat-sensitive function necessary for viral replication (Gharpure, 1965).

In view of the well documented effect of heat on the pattern of protein synthesis, it was of interest to determine if protein synthesis was necessary during the induction period for the eventual expression of HER as has been demonstrated for RER (Das Gupta & Summers, 1978; Lytle, 1978). The protein synthesis inhibitor cycloheximide was used to investigate this directly.

At various times after heat shock, cycloheximide was added to the growth medium, the cells incubated for a total of 36 h, the cycloheximide removed and the irradiated virus added. Figure 5 shows that cycloheximide has little effect on HER when added any time after 3 h post-shock. However if added during the first 3 h of post-treatment incubation, subsequent HER was substantially reduced. This suggests that, in addition to some process which takes up to 36 h for full expression, HER is dependent on protein synthesis immediately after heating. The UV-enhanced reactivation of UV-irradiated herpes simplex virus in CV-1 monkey kidney cells has been shown to require protein synthesis during the first 6–8 h of post-irradiation incubation although a 24 h delay between irradiation and infection gave optimum reactivation (Lytle & Goddard, 1979).

**Figure 5** Effect of adding cycloheximide at different times after heat shock on the reactivation factor of irradiated virus. After heating the cells for 10 min at 45.5°C, cycloheximide (10 $\mu$g ml$^{-1}$) was added at various times during the 36 h post-heating period and the cells infected with irradiated or unirradiated virus in the absence of cycloheximide. Points represent the means ($\pm$1 s.d.) of three determinations.

### Discussion

The exposure of HeLa cells to a brief hyperthermic shock enhances their ability to reactivate and support the replication of UV-irradiated adenovirus 2 by almost one order of magnitude. Maximum reactivation is achieved by heating the cells for 10 min at 45.5°C and by delaying the infection for 36 h.

It has previously been shown that heating BHK or HeLa cells at 45°C for 15 min reduces the infectivity of DNA viruses to ~10% (Gharpure, 1965), suggesting that a heat-sensitive host function is necessary for the replication of such viruses. The high infectivity of unirradiated virus which we find here is probably a result of the briefer heat treatment and the delay between heating and infection. The latter factor would allow time for recovery of the heat-sensitive function. Nevertheless it is still possible that the 3-fold enhancement of reactivation observed upon immediate infection is an underestimate of the efficiency of the induced response at that time due to persisting thermal damage. This may explain the apparently longer time required for the development of maximum HER (36 h) compared to RER (18 h).

The requirement for early protein synthesis suggests a possible role for the HeLa heat-shock proteins in this response since their rate of synthesis is maximal 2 h after heating. Furthermore they are fully induced by a 5 min heat shock at 45°C (Slater *et al.*, 1981; Burdon *et al.*, 1982). Those agents such as UV-irradiation and mitomycin C which are known to induce RER have also been shown to enhance the synthesis of specific proteins (Herrlich *et al.*, 1982). It will be of interest to see whether these proteins represent a subset of the heat-shock proteins.

Nevins (1982) has reported that infection of HeLa cells with adenovirus 5 leads to the induction of the human 70 K dalton heat-shock protein, an effect mediated by the viral E1A gene product. It might be suggested therefore that HER is the result of a more efficient infection due to the initial presence of this heat-shock protein in the heated cells. However adenovirus 5 produces no more plaques on 293 cells, human embryonic kidney cells transformed by the left end of the adenovirus 5 genome and which constitutively express the viral E1A gene and the cellular 70 K dalton heat-shock protein gene, than it does on HeLa cells (Graham *et al.*, 1977).

As the properties of HER reported here are similar to those of the well characterised RER observed with many cell/virus systems, it is possible that the biochemical mechanisms of the two reactivation responses have at least some common components; heat may therefore be an alternative trigger for the induction of this response. A consequence which heating shares with those agents which induce RER and which may be a common trigger is the inhibition of DNA synthesis (Warters & Stone, 1983). Experiments are in progress to investigate this possibility and to study the ability of chemicals such as ethanol, $Cu^{2+}$ and arsenite, which induce some or all of the heat-shock proteins (Burdon *et al.*, 1982), to elicit the HER response.

The use of UV-irradiated virus as a sensitive probe for cellular repair functions has demonstrated the existence of a heat-enhanced DNA repair or lesion bypass mechanism. If, as seems likely, HER also operates upon ionising radiation-induced DNA damage, it may be an important consideration in the combined use of hyperthermia and radiation in cancer therapy. If induced by an initial hyperthermic treatment in a clinical fractionated dose regimen, such a mechanism could conceivably reduce the efficiency of subsequent radiotherapy whose principal intracellular target is DNA. Furthermore, evidence has been presented that RER may be accompanied by enhanced mutagenesis though this may not always be the case

(DasGupta & Summers, 1978; Sarasin & Benoit, 1980; Day & Ziolkowski, 1981; Lytle & Knott, 1982; Taylor *et al.*, 1982). If HER were also mutagenic, this could represent an undesirable side effect of the irradiation of heated healthy tissue which would compromise the possible value of using thermotolerance as a means of preferentially protecting normal tissue (Hahn, 1982). It remains to be established whether or not HER of adenovirus 2 in HeLa cells involves a mutagenic component.

The authors are indebted to the Cancer Research Campaign for support and to Dr W.C. Russell and Dr V. Mautner for assistance in establishing the methods for virus growth and plaque assay.

### References

ADAMS, R.L.P. (1980). *Cell Culture for Biochemists*. Amsterdam: Elsevier North Holland, p. 62.

BOCKSTAHLER, L.E. (1981). Induction and enhanced reactivation of mammalian viruses by light. *Prog. Nucl. Acid Res. Mol. Biol.*, **26**, 303.

BOCKSTAHLER, L.E. & LYTLE, C.D. (1977). Radiation enhanced reactivation of nuclear replicating mammalian viruses. *Photochem. Photobiol.*, **25**, 477.

BURDON, R.H., SLATER, A., McMAHON, M. & CATO, A.C.B. (1982). Hyperthermia and the heat-shock proteins of HeLa cells. *Br. J. Cancer*, **45**, 953.

CORRY, P.M., ROBINSON, S. & GETZ, S. (1977). Hyperthermic effects on DNA repair mechanisms. *Radiology*, **123**, 475.

DASGUPTA, U.B. & SUMMERS, W.C. (1978). Ultraviolet reactivation of herpes simplex virus is mutagenic and inducible in mammalian cells. *Proc. Natl Acad. Sci.*, **75**, 2378.

DAY, R.S. III. (1977). Human adenoviruses as DNA repair probes. In: *DNA Repair Processes (*Eds., Nichols & Murphy). Miami: Symposia Specialists Inc. p. 119.

DAY, R.S. III & ZIOLKOWSKI, C.H.J. (1981). U.v.-induced reversion of adenovirus 5ts2 infecting human cells. *Photochem. Photobiol.*, **34**, 403.

DEWEY, W.C., HOPWOOD, L.E., SAPARETO, S.A. & GERWECK, L.E. (1977). Cellular responses to combinations of hyperthermia and radiation. *Radiology*, **123**, 463.

DEWEY, W.C., FREEMAN, M.L., RAAPHORST, G.P. & 7 others. (1980). Cell biology of hyperthermia and radiation. In: *Radiation Biology in Cancer Research* (Eds. Meyn & Withers). New York: Raven Press. p. 589.

GERNER, E.W. & SCHNEIDER, M.J. (1975). Induced thermal resistance in HeLa cells. *Nature*, **256**, 500.

GHARPURE, M.A. (1965). A heat-sensitive function required for the replication of DNA viruses but not RNA viruses. *Virology*, **27**, 308.

GRAHAM, F.L., SMILEY, J., RUSSELL, W.C. & NAIRN, R. (1977). Characteristics of a human cell line transformed by DNA from human adenovirus type 5. *J. Gen. Virol.*, **36**, 59.

HAHN, G.M. (1982). *Hyperthermia and Cancer*. New York: Plenum Press.

HAHN, G.M. & LI, G.C. (1982). Thermotolerance and heat shock proteins in mammalian cells. *Radiat. Res.*, **92**, 452.

HENLE, K.J. & DETHLEFSEN, L.A. (1978). Heat fractionation and thermotolerance: a review. *Cancer Res.*, **38**, 1843.

HERRLICH, P., RAHMSDORF, H.J., MALLICK, U. & 4 others. (1982). Radiation and mutagen inducible mammalian genes. *Biochimie*, **64**, 707.

LYTLE, C.D. (1978). Radiation-enhanced reactivation in mammalian cells. *Natl Cancer Inst. Monogr.*, **50**, 145.

LYTLE, C.D. & GODDARD, J.G. (1979). U.v.-enhanced virus reactivation in mammalian cells: effects of metabolic inhibitors. *Photochem. Photobiol.*, **29**, 959.

LYTLE, C.D. & KNOTT, D.C. (1982). Enhanced mutagenesis parallels enhanced reactivation of herpes virus in a human cell line. *EMBO J.*, **1**, 701.

MEZZINA, M., GENTIL, A. & SARASIN, A. (1981). Simian virus 40 as a probe for studying inducible repair functions in mammalian cells. *J. Supramol. Struct. Cell. Biochem.*, **17**, 121.

NEVINS, J.R. (1982). Induction of the synthesis of a 70,000 dalton mammalian heat-shock protein by the adenovirus E1A gene product. *Cell*, **29**, 913.

RADMAN, M. (1980). Is there SOS induction in mammalian cells? *Photochem. Photobiol.*, **32**, 823.

SARASIN, A. & BENOIT, A. (1980). Induction of an error-prone mode of DNA repair in u.v.-irradiated monkey kidney cells. *Mutat. Res.*, **70**, 71.

SCHLESINGER, M.J., ASHBURNER, M. & TISSIERES, A. (1982). *Heat Shock from Bacteria to Man*. New York: Cold Spring Harbor Laboratory.

SLATER, A., CATO, A.C.B., SILLAR, G.M., KIOUSSIS, J. & BURDON, R.H. (1981). The pattern of protein synthesis induced by heat shock of HeLa cells. *Eur. J. Biochem.*, **117**, 341.

SPIRO, I.J., DENMAN, D.L. & DEWEY, W.C. (1982). Effect of hyperthermia on CHO DNA polymerases $\alpha$ and $\beta$. *Radiat. Res.*, **89**, 134.

TAKIMOTO, K., NIWA, O. & SUGAHARA, T. (1982). Reactivation of u.v.-and $\gamma$-irradiated herpes virus in u.v.- and X-irradiated CV-1 cells. *Photochem. Photobiol.*, **35**, 495.

TAYLOR, W.D., BOCKSTAHLER, L.E., MONTES, J., BABICH, M.A. & LYTLE, C.D. (1982). Further evidence that ultraviolet radiation-enhanced reactivation of simian virus 40 in monkey kidney cells is not accompanied by mutagenesis. *Mutat. Res.*, **105**, 291.

VOS, J.M., CORNELIS, J.J., LIMBOSCH, S., ZAMPETTI-BOSSELER, F. & ROMMELAERE, J. (1981). U.v.-irradiation of related mouse hybrid cells: similar increase in capacity to replicate intact minute-virus-of-mice but differential enhancement of survival of u.v.-irradiated virus. *Mutat. Res.*, **83**, 171.

WARTERS, R.L. & ROTI ROTI, J.L. (1982). Hyperthermia and the cell nucleus. *Radiat. Res.*, **92**, 458.

WARTERS, R.L. & STONE, O.L. (1983). The effects of hyperthermia on DNA replication in HeLa cells. *Radiat. Res.*, **93**, 71.

WILLIAMS, J.F. (1970). Enhancement of adenovirus plaque formation on HeLa cells by magnesium chloride. *J. Gen. Virol.*, **9**, 251.

WITKIN, E.M. (1976). Ultraviolet mutagenesis and inducible DNA repair in *Escherichia coli*. *Bacteriol. Rev.*, **40**, 869.